# Cross-Generational Effects and Non-random Developmental Response to Temperature Variation in *Paramecium*

**DOI:** 10.3389/fcell.2020.584219

**Published:** 2020-10-20

**Authors:** Rebecca Hagen, Valerio Vitali, Francesco Catania

**Affiliations:** Department of Biology, Institute for Evolution and Biodiversity, University of Münster, Münster, Germany

**Keywords:** programmed DNA elimination, adaptive developmental plasticity, temperature, genome evolution, *Paramecium*, intergenerational effects, epigenetics

## Abstract

Unicellular organisms such as ciliates are largely neglected in research on adaptive developmental plasticity, although their nuclear dualism offers ideal circumstances to study development outside an embryonic context. Here, we gain first insights into the ability of the ciliate *Paramecium* to develop potentially adaptive phenotypic changes in response to early-life adversity. We show that, upon exposure to unconventional culture temperatures, germ line-to-soma differentiation gives rise to coordinated molecular changes that may help attune the number of functional gene copies to the new external conditions. The non-random somatic heterogeneity that developmental plasticity generates is largely epigenetically controlled, shaped by the parental experience, and may prompt a stress response. These findings establish *Paramecium* as a new model system to study the molecular basis and evolutionary significance of developmental plasticity. In echoing previous indications in mammals, they call for an incorporation of intergenerational effects in adaptation studies.

## Introduction

Environmental cues affecting organismal development may produce long-term patterns of gene expression and induce novel phenotypes as life advances (Beldade et al., 2011; Cole et al., 2012; Tung et al., 2015). These phenotypes may or may not be associated with disease, and may be passed on to the next sexual generation(s) via epigenetic mechanisms. Thus, developmental plasticity (henceforth referred to as plasticity) may contribute both to diseases and the onset of evolutionary adaptations. Despite the seemingly straightforward ways of testing this possibility, whether and how plasticity truly affects health and/or evolution are still matters of contention.

Existing models on the role of plasticity in health and evolution make different, even conflicting predictions. Regarding health, plasticity may either enhance survival in early life at the expense of adult health (developmental constraints model), or prepare an individual to respond optimally to an anticipated environment later in life (predictive adaptive response model) (Nettle and Bateson, 2015; Lea et al., 2017). Both developmental constraints and predictive models assume that plasticity evolved through natural selection (Watve, 2017). However, only the predictive model seems to maintain that individuals that are exposed to low-quality environments in early life will respond adaptively later in life by maximizing reproduction within their expected shorter lifespan (Wells, 2012). As for the significance of plasticity in evolution, evidence from natural populations suggests that adaptive and non-adaptive plasticity can both hinder and facilitate adaptation to new environments (e.g., Ghalambor et al., 2015) vs. (Scoville and Pfrender, 2010). These seemingly contradictory observations build on substantial theoretical research and a relatively small number of empirical studies, an insufficiency that is, in part, attributable to historical skepticism toward the significance of plasticity for evolution (Sommer, 2020). Experimental evolution studies in natural settings may be viewed as the best way to obtain valuable insights into how evolution proceeds in nature. However, these studies have also some disadvantages. For example, they may occur in complex ecosystems and unfold over long time periods, circumstances that may make it difficult to control for all relevant ecological factors contributing to selection pressures. New tractable model systems where the evolutionary significance of plasticity can be easily and rapidly tested under controlled laboratory conditions would be highly valuable to evolutionary biologists. These systems may help in identifying conserved molecular mechanisms that influence developmental reprogramming in response to the environment, and provide insights into how to integrate the roles that plasticity plays in health and evolution.

**FIGURE 1:**
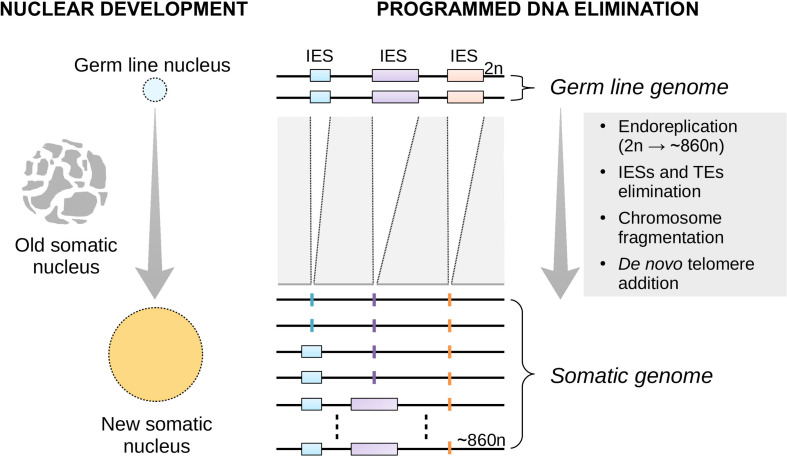
Nuclear replacement and somatic genome development in *Paramecium*. Each time the single-celled *Paramecium* reproduces sexually, the old (parental) somatic nucleus is destroyed and replaced with a new one **(left)**. The new macronucleus develops from a copy of the zygotic micronucleus housing the germ line genome. The process of genome remodeling that accompanies nuclear development in *Paramecium* involves endoreplication, chromosome fragmentation and *de novo* telomerization, and the elimination of mostly germ line-restricted elements such as transposable elements (TEs) and Internal Eliminated Sequences (IESs) **(right)**. Only IES elimination is shown. This developmentally-regulated genome processing is known as Programmed DNA Elimination (PDE). PDE is crucial to reconstitute a functional somatic genome from an interrupted germ line template.

To date, virtually all of the studies on plasticity have focused on multicellular organisms (Gluckman et al., 2009; Beldade et al., 2011; Bateson et al., 2014; Lea et al., 2017; Lu et al., 2019). This is because *development* is commonly viewed as an embryonic or cellular process. However, development may also take place at the sub-cellular level. Ciliates such as the free-living *Paramecium tetraurelia* (henceforth referred to as *Paramecium*) offer a spectacular example of nuclear development. *Paramecium* contains a diploid germ line (micro)nucleus and a highly polyploid somatic (macro)nucleus in a single cell. Unlike the germ line micronucleus, the somatic macronucleus is transcriptionally active during the cell’s vegetative life and determines its phenotype. At each sexual cycle, the macronucleus degrades slowly and a new (filial) somatic macronucleus differentiates from a mitotic copy of the zygotic nucleus (Coyne et al., 1996, 2012; Betermier and Duharcourt, 2014) (Figure 1).

It has become increasingly clear that *Paramecium* biology is close to that of many other eukaryotes. For example, sex, stress response, growth, and lifespan are mechanistically linked in *Paramecium* as they are in multicellular eukaryotes (Arking, 2018). For example, low-quality food accelerates sexual maturation in *Paramecium* (Thind et al., 2020), and the timing of sexual maturity in this microbial eukaryote positively correlates with lifespan (Smith-Sonneborn, 1981). At the same time, *Paramecium* possesses biological properties that are conveniently different from those of multicellular and long-lived organisms that are commonly used to investigate adaptive plasticity. Indeed, as *Paramecium* can be grown in the lab for successive sexual generations without essentially acquiring new germ line mutations (Sung et al., 2012; Long et al., 2018), the short- and long-term effects of plasticity on the fitness of isogenic lines can be readily distinguished and assessed. All of this makes *Paramecium* an ideal system by which to advance general understanding of the complex interplay between epigenetics and genetics and to generate hypotheses on the effect of development on adaptation that can be tested in less amenable systems.

Furthermore, empirical findings in *Paramecium* might help further advance current understanding of plasticity in multicellular systems. In one example, studies on *Paramecium* could help assess whether adaptive plasticity is only triggered in response to environments that the organism has recurrently experienced over evolutionary time. Studies on *Paramecium* could also expand current views and generate fresh hypotheses in metazoans. For example, the timing of sexual maturity in *Paramecium* is reduced not only by the low quality of the environmental conditions (Thind et al., 2020) but also as the age of the parental cells increases (Siegel, 1961; Takagi et al., 1987). Thus, in addition to early life environments, parental experience may affect the phenotypic state of an individual. Incorporating parental effects in current explanations for plasticity in metazoans is essential to gain a more accurate perspective of the role of plasticity in evolution (Wells, 2017).

Nuclear development in *Paramecium* generates molecular variation (Duret et al., 2008; Catania et al., 2013), more so when *Paramecium* faces new environmental conditions (Vitali et al., 2019). This somatic genome plasticity is, at least in part, the byproduct of a perturbed developmental process known as programmed DNA elimination (PDE). In *Paramecium*, PDE operates at the genome-wide level and eliminates ∼45,000 intervening DNA segments—Internal Eliminated Sequences (IESs)—during the development of a new somatic macronucleus (Arnaiz et al., 2012) (Figure 1). Although this elimination is largely faithful, PDE may fail to remove some IESs from all the copies of the newly developing somatic genome. Thus, 100s of *Paramecium* IESs may accumulate in the new somatic macronucleus at each sexual cycle, with a variable fraction of somatic DNA copies affected by the IES incorporation.

Incomplete IES excisions are commonly regarded as errors with limited biological significance. However, previous studies have found signatures of purifying selection antagonizing IES retention (Arnaiz et al., 2012; Ferro et al., 2015; Vitali et al., 2019). This suggests that incomplete IES excision in *Paramecium* may have non-trivial phenotypic consequences. Furthermore, although the somatic nucleus is replaced at each sexual cycle, somatic IESs may nonetheless be transmitted to the sexual offspring via RNA-mediated epigenetic mechanisms (Duharcourt et al., 1995, 1998, 2009). These mechanisms are similar to those that contribute to the trans-generational transmission of parental phenotypic responses in animals, fungi, and plants (Duempelmann et al., 2020). Thus, epigenetic mechanisms in *Paramecium* may enable the inheritance of somatic IESs and their potential phenotypic effects—these changes might later become genetically encoded. Further, conserved RNA-mediated molecular dynamics may underlie the evolutionary significance of plasticity across developmental systems, bridging the gap between ciliates and metazoans.

Here, we consider the process of germ line to soma differentiation in the single-celled *Paramecium* and characterize the relationship between this developmental process’s plasticity and somatic gene expression (a proxy for phenotypic variation). We uncover coordinated and predictable somatic changes, which allow causally linking the environments that *Paramecium* experiences during its vegetative life/somatic development to the number of productive somatic gene copies in its sexual offspring. In extending our previous work (Vitali et al., 2019), these findings empirically connect ecology and development in a microbial eukaryote while offering insights into the molecular processes that underlay plasticity.

## Materials and Methods

### *Paramecium* Stocks, Culture Conditions, Published and Newly Generated Genome Datasets

**FIGURE 2:**
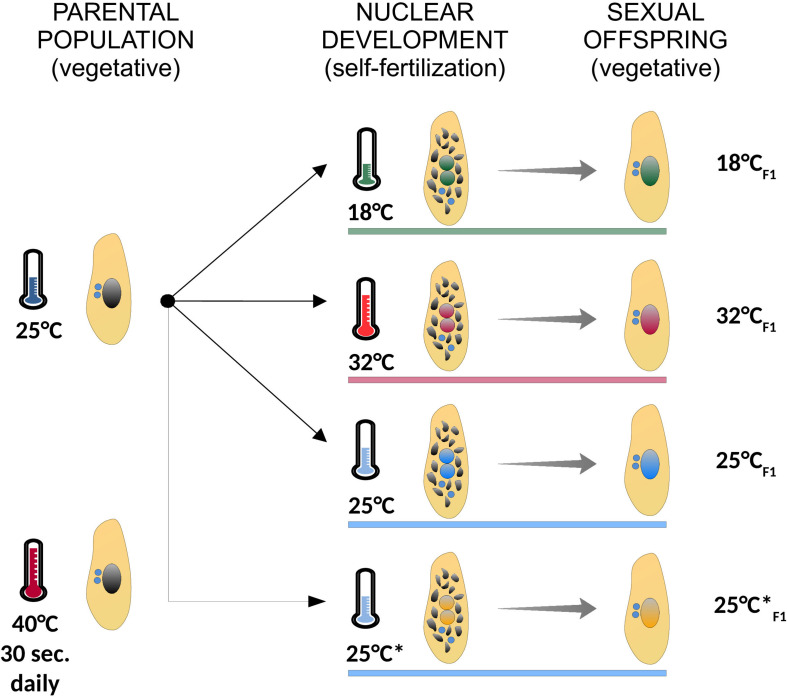
Summary of the experimental treatments used in the study. A fully clonal population of paramecia (identical germ line and somatic genomes) was cultured at 25°C, and subjected to different environmental temperatures during self-fertilization (18°C, green; 32°C, red; 25°C, blue; 25°C*, orange), when somatic genome development takes place. In the following vegetative phase, when cells divide asexually, the ensuing sexual offspring was expanded to mass culture for genomic DNA sequencing. The parental population from which Line 25°C*_F1_ originates was subjected to a daily 40°C-heat shock for 30 s. Cells were grown at the indicated experimental temperature (colored horizontal bars) both during nuclear development (self-fertilization) and the ensuing vegetative phase (sexual offspring). Asexually dividing cells are depicted with a large intact somatic nucleus (black in the parental population and colored by treatment in the sexual offspring). Self-fertilizing cells are depicted during nuclear development with two developing nuclei (with colors matching the treatment) and the old somatic nucleus on its way to be fully degraded (multiple black fragments). Germ line nuclei are depicted in blue.

We re-examined the IES excision profiles of fully homozygous lines of *P. tetraurelia* stocks 51 and d12, which were generated in previously published experiments (Arnaiz et al., 2012; Lhuillier-Akakpo et al., 2014; Swart et al., 2017; Vitali et al., 2019). *P. tetraurelia* stocks 51 cells were cultured at 27°C during both vegetative growth and nuclear differentiation, when somatic genome development/Programmed DNA Elimination takes place (Arnaiz et al., 2012; Lhuillier-Akakpo et al., 2014; Swart et al., 2017). A fully clonal population of *P. tetraurelia* stocks d12 was cultured at 25°C (F0) and subjected to 18, 25, or 32°C (F1) during somatic genome development and the successive vegetative phase, when cells divide asexually. These lines were generated in Vitali et al. (2019) to investigate the impact of different environmental temperatures on somatic genome development. One additional F1 line (referred to as 25°C^∗^_*F*1_ in the main text) was also generated in Vitali et al. (2019), but analyzed here for the first time. Line 25°C^∗^_*F*1_ originates from the same parental population that fathered lines 18°C_*F*1_, 25°C_*F*1_, and 32°C_*F*1_ and like 25°C_*F*1_ was subjected to 25°C during nuclear differentiation. However, the parental cells of 25°C^∗^_*F*1_ were additionally subjected to a 40°C heat shock for 30 s daily during their vegetative life. Line 25°C^∗^_*F*1_ and 25°C_*F*1_ were used to test the impact of ecological factors experienced by the parental population on a future instance of somatic genome development. The set of treatments applied to the fully clonal parental population of *P. tetraurelia* stock d12 are summarized in Figure 2. For each of the lines surveyed, isogenic cells were expanded to mass culture for somatic DNA extraction (as previously described Arnaiz et al., 2012; Lhuillier-Akakpo et al., 2014; Swart et al., 2017; Vitali et al., 2019). Culture conditions, macronuclear DNA isolation, whole-genome sequencing and data preprocessing for the 25°C^∗^_*F*1_ sample are as previously described (Vitali et al., 2019).

### IES Datasets Analyses

The software ParTIES (Denby Wilkes et al., 2016) was used to estimate IES Retention Scores (IRSs), i.e., the *per*-locus ratio between IES-containing reads and the total number of mapping reads. IES loci with significantly different retention levels between F0 and F1 lines were designated as previously described (Vitali et al., 2019). Namely, the upper and lower bound of the 75% confidence interval constructed on the F0 retention score was taken as a reference retention score for binomial testing of upward or downward transitions, respectively. IES loci supported by < 20 sequence reads were excluded and only IESs with a size larger than 25 nucleotides are considered for this study. The sets of IESs whose excision is epigenetically controlled in *P. tetraurelia* stock 51 were obtained from ParameciumDB (Arnaiz and Sperling, 2011).

### Gene Expression Data and GO Term Enrichment

Transcriptomes were previously obtained for *P. tetraurelia* stocks 51 (Arnaiz et al., 2017) and d12 (Vitali et al., 2019), using cells at the vegetative stage cultured at standard conditions (25–27°C). High and weak gene expression level categories are based on whether the expression values fall into the first or the last quartile of an underlying log2-transformed distribution. The functional enrichment analyses of somatic-IES containing genes were performed using the functional annotation tool DAVID (Huang et al., 2009a,b). As reference set for the genes that harbor DCL2/3-sensitive, PTC-inducing, and exon-mapping small IESs and are highly expressed at standard conditions, we used genes that harbor small exon-mapping IESs. As reference set for the genes that are weakly expressed at standard conditions and that harbor DCL5-sensitive, PTC-inducing, large and exon-mapping IESs, we used genes that harbor large exon-mapping IESs. In all cases, Fisher’s exact test estimates the over-representation of GO-terms.

### Data Analysis

All the analyses were conducted using R Core Team (2018).

### Data Access

High-throughput sequencing data generated for the 25°C^∗^_*F*1_ sample have been submitted to the European Nucleotide Archive^1^ under the accession number ERR4179861.

## Results and Discussion

### A Relationship Between PDE Efficiency and Somatic Gene Expression Level

We asked whether vegetative gene expression levels and PDE-mediated developmental variation are linked in *Paramecium*. PDE removes Internal Eliminated Sequences (IESs) from the developing polyploid somatic genome and this removal can be incomplete. The *per*-locus magnitude of somatic IES retention is approximated by the relative fraction of IES-containing sequence reads and is termed IES Retention Score (IRS = 0, no retention; IRS = 1, full retention).

Using previously published transcriptomic data (Arnaiz et al., 2017) and two independent sets of incompletely excised IESs from the same *Paramecium* stock cultivated in standard conditions (Arnaiz et al., 2012; Lhuillier-Akakpo et al., 2014), we find that gene expression levels correlate negatively with IRS estimates (Kendall’s *tau* ≤ −0.127, *P* < 0.0001). The high statistical significance of this observed negative relationship between IRS and gene expression levels holds when single-copy genes and genes with duplicates are examined separately. As differently sized IESs may be subjected to different mechanisms of excision (Arnaiz et al., 2012; Ferro et al., 2015), we checked whether the statistical significance of this correlation holds for small and large IESs alike. Additionally, we inspected the distribution of non-trivial IES retentions (IRS > 0.1) in genes that are weakly and highly expressed (WEG and HEG, respectively). We found that WEG harbor a statistically significant excess of somatic IESs relative to HEG (Table 1). This excess holds firmly for large IESs, but it is less pronounced for small IESs (Table 1). Furthermore, small IESs undergo > 3 times more incomplete excision than large IESs in HEG (0.25 vs. 0.08%), whereas they display comparable levels of incomplete excision to large IESs in WEG (0.84 vs. 0.97%, respectively). Thus, the level of developmental variation in *Paramecium* varies with the level of somatic gene expression, and the strength of this relationship is IES-size dependent.

**TABLE 1:** Incomplete IES excision (IRS > 0.1) affects weakly expressed genes (WEGs) more frequently than highly expressed genes (HEGs).

IESs	IESs in HEG (IRS ≥ 0)	IESs in WEG (IRS ≥ 0)	IESs in HEG (IRS > 0.1)	IESs in WEG (IRS > 0.1)	Two-proportion Z-test, P*
All	8108	9504	12	87	2.3 e-11
Large	5142	4725	4	46	9.5 e-10
Small	2765	4544	7	38	0.0033

The underlying distribution of IESs (IRS ≥ 0) is based on the IES dataset generated in Lhuillier-Akakpo et al. (2014). IESs with IRS > 0.1 are incompletely excised either in the experiment LA (Lhuillier-Akakpo et al., 2014) or in the experiment AR (Arnaiz et al., 2012). Partitioning of IESs based on size class reveals large IESs (>45 bp) contribute to this excess in WEG more substantially than small IESs (26–30 and 44–45 bp). Additionally, small IESs undergo incomplete excision more often than large IESs in HEGs. IESs were partitioned in size groups following (Ferro et al., 2015). *No correction for multiple testing.

### Natural Selection Affects IES Distribution and Splicing Fidelity in the *Paramecium* Genome

Two observations can help explain why WEG retain an excess of large IESs (Table 1). First, WEG in *Paramecium* experience lower levels of selective pressure compared to HEG (Gout et al., 2010). Second, large WEG-mapping IESs display weaker *cis*-acting DNA-level splicing signals compared to same-size HEG-mapping large IESs (0.60 vs. 0.64; Wilcoxon rank sum test, *P* = 0.023). Thus, the relatively lower selective pressure on WEG may contribute to the deterioration of large IESs’ *cis*-acting recognition/excision signals, thereby increasing these IESs’ risk of incomplete excision. As small IESs may rely less on *cis*-acting sequence signals compared to large IESs (Ferro et al., 2015), this explanation also accounts for the marginal over-representation of small somatic IESs in WEG (Table 1).

Rationalizing the threefold surplus of incompletely excised small IESs in HEG is less straightforward. If incomplete IES excision reflects errors and may be detrimental as commonly postulated, then natural selection is expected to purge small IESs from HEG. Consistent with this, there is a deficit of small IESs in *Paramecium* HEG (Ferro et al., 2015 and Table 1). But then, it is unclear why a subset of small IESs that undergo potentially hazardous incomplete elimination is found in HEG.

One possibility is that modern HEG-mapping small IESs undergo only trivial and thus tolerable levels of retention. The data at our disposal do not align well with this scenario. As illustrated in Table 1, the incomplete excision of HEG-mapping small IESs may entail > 10% of the somatic DNA copies (IRS > 0.1). Additionally, we detect a systematically higher number of small IESs with IRS > 0.1 in HEG relative to large IESs in each of the two experiments considered above and in two other independent experiments that we revisited (Swart et al., 2017) (in total: 15 vs. 6, respectively).

Another possible explanation for the evolutionary persistence of small IESs in HEG is that their retention has no impact on protein sequence/function. However, upon examining four independent experiments (Arnaiz et al., 2012; Lhuillier-Akakpo et al., 2014; Swart et al., 2017), we detect a significant enrichment (rather than a deficit) of premature termination codons (PTCs) induced upon retention of small IESs compared to what would be expected by chance (1.1 vs. 0.5%, *P* = 0.009). We find no statistically significant enrichment in WEG instead (3.0 vs. 2.6%, *P* = 0.281) (note that in this latter analysis we used IESs with IRS > 0.05, rather than IRS > 0.1, to increase the power of the statistical test).

In sum, our observations raise the possibility that natural selection might actually favor the incomplete excision of a subset of small and PTC-inducing IESs in a variable fraction of HEG. The question then arises: is the alternative splicing of these IESs in *Paramecium*’s HEG functional?

### The Count of Incompletely Excised Small Exon-Mapping IESs Increases Upon Environmental Changes

*Paramecium* genes that retain PTC-inducing introns generate transcripts that normally become targets of the Nonsense Mediated Decay (NMD) pathway for degradation (Jaillon et al., 2008). Thus, the partial retention of small PTC-inducing IESs in *Paramecium*’s HEG may effectively reduce the number of productive (i.e., functional) gene copies in the somatic nucleus. This may impact the levels of somatic transcription, thereby helping rationalize the detected relationship between PDE-mediated developmental variation and somatic gene expression levels. An IES-mediated regulatory function might also help *Paramecium* cope with environmental changes. More specifically, environmental changes during somatic development could affect the excision of small PTC-inducing IESs tuning the number of productive somatic gene copies to the new surroundings. This hypothetical scenario predicts that small HEG-mapping IESs that introduce PTCs upon incomplete excision will enter into play when *Paramecium* is exposed to new environments.

**FIGURE 3:**
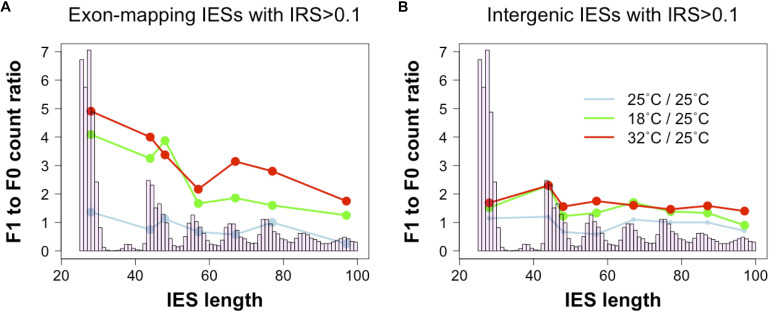
Ratio of incomplete IES excision (IRS > 0.1) counts in parental and filial generations. The two consecutive generations are exposed to either a stable environment in which both F0 and F1 were cultivated at 25°C (light blue) or a changing environment in which the F0 was cultivated at 25°C, but the F1 was cultivated either at 18°C (green) or 32°C (red). Grouping IESs according to genomic location (coding exons, intergenic) reveals that small exon-mapping IESs are more often incompletely excised compared to larger exon-mapping IESs **(A)**. No size-dependent pattern is detected for intergenic IESs **(B)**. Ratios were calculated after summing incompletely excised IESs falling in the most frequent size classes (bp): 26–30, 44–45, 46–50, 54–60, 64–70, 74–80, 84–90, and 94–100. Only size classes with ≥ 3 incompletely excised IESs *per* condition were examined.

To test this hypothesis, we revisited a dataset generated recently (Vitali et al., 2019). We examined changes in incomplete IES excision across two consecutive sexual generations (F0 and F1) with or without changes in environmental temperature (25°C_*F*0_ → 25°C_*F*1_; 25°C_*F*0_→ 18°C_*F*1_; 25°C_*F*0_ → 32°C_*F*1_). We find that F0 and F1 cells that are both cultivated at 25°C display comparable numbers of small and large somatic IESs (F1/F0 ratio: 1.3 and 0.8 for small IESs and large IESs, respectively). However, significant differences emerge when we consider F0 and F1 cells that are exposed to different temperatures. The count of somatic IESs in the 18°C_*F*1_ and 32°C_*F*1_ lines not only rises considerably, as previously described (Vitali et al., 2019), but is also unevenly distributed with regard to IES size and gene expression levels. Relative to the cells cultivated at 25°C, 18°C_*F*1_, and 32°C_*F*1_ cells contain more somatic small IESs (4 and 5 times, respectively) than large IESs (2 and 2.5 times, respectively) (Figure 3A). These IES size-dependent differences disappear for IESs that map to intergenic regions (Figure 3B). Furthermore, small somatic IESs in 32°C_*F*1_ cells preferentially reside in genes that are highly expressed in the parental cells (two-proportion Z-test, *P* = 0.006)—we detected a similar preference for 25°C_*F*1_ (two-proportion Z-test, *P* = 0.05). More than 70% of these IESs (11/15) introduce in-frame stops based on *P. tetraurelia* stock 51’s gene annotation.

These observations support a tentative model where IES-mediated gene copy disruption mediates the down-regulation of a subset of *Paramecium* genes.

### The Magnitude of Incomplete Excision for Large Exon-Mapping IESs Decreases Upon Environmental Changes

**FIGURE 4:**
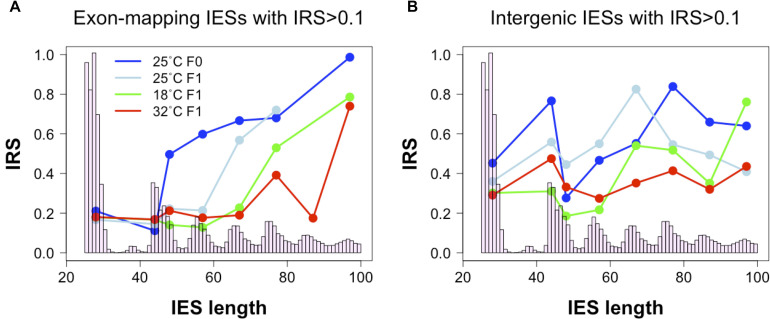
Relationship between IES size and IES Retention Score (IRS). **(A)** The median IRS of small IESs, but not of large IESs, is comparable between F0 and F1 cells cultivated at 25°C (dark and light blue lines) and at the non-standard culture temperatures of 18°C (green line) and 32°C (red line). **(B)** In IESs that map to intergenic regions no clear IES size-related trend is discernible. Median IRS values were calculated using incompletely excised IESs that fall in the most frequent size classes (bp): 26–30, 44–45, 46–50, 54–60, 64–70, 74–80, 84–90, and 94–100. Only size classes with ≥ 3 incompletely excised IESs *per* condition were examined.

In the course of our analyses, we also detected a positive correlation between IES retention levels and the size of exon-mapping IESs with IRS > 0.1 at 25°C (Figure 4A, 25°C_*F*0_: Kendall’s *tau* = 0.188, *P* = 0.009; 25°C_*F*1_: Kendall’s *tau* = 0.169, *P* = 0.019). Additionally, the average IRS of large exon-mapping IESs is significantly reduced in 18°C_*F*1_ and 32°C_*F*1_ cells compared to the parental cells cultivated at 25°C (Wilcoxon rank sum test, *P* < 0.0001). Finally, F1 cells cultured at 18 and 32°C tend to accumulate better-excised, large and exon-mapping IESs in genes that are weakly expressed in the parental environment (18°C_*F*1_: 5 large vs. 2 small IESs with reduced IRS; 11 large vs. 21 small IESs with increased IRS; Fisher’s exact test, *P*_*one–tail*_ = 0.085; 32°C_*F*1_: 7 large vs. 2 small IESs with reduced IRS; 25 large vs. 35 small IESs with increased IRS; Fisher’s exact test, *P*_*one–tail*_ = 0.047).

These observations raise the possibility that the hypothetical IES-mediated regulation of productive somatic gene copy number in *Paramecium* may proceed not only through gene copy disruption (via small IES retention in HEG), but also through gene copy re-activation (via large IES excision from WEG). This large IES-mediated regulation may extend to intergenic regions where significant changes in IRS are recorded both for large and small intergenic IESs at 32°C (Wilcoxon rank sum test, *P* < 0.005) and for small IESs at 18°C (Wilcoxon rank sum test, *P* = 0.011) (Figure 4B).

### IES-Mediated Changes in Functional Gene Copies May Be Epigenetically Controlled

Because *cis*-acting IES recognition/excision signal sequences are highly likely to remain invariable across two consecutive sexual generations, *trans*-acting factors must shape the aforementioned differences in small and large IES recognition/excision efficiency. We therefore examined how extensively the putative regulated/regulatory IESs fall into a class of IESs that are under epigenetic control in the *P. tetraurelia* stock 51 (henceforth referred to as epi-IESs). Two classes of small RNAs are involved in the excision of epi-IESs in *Paramecium*. Two Dicer-like proteins (DCL2 and DCL3) produce scnRNAs that transit through the parental somatic nucleus from the germ line nucleus before targeting IESs for excision in the developing somatic nucleus (Lepere et al., 2009; Bouhouche et al., 2011). The Dicer-like protein DCL5 produces iesRNAs directly in the developing somatic nucleus from excised amplified IESs (Sandoval et al., 2014).

**FIGURE 5:**
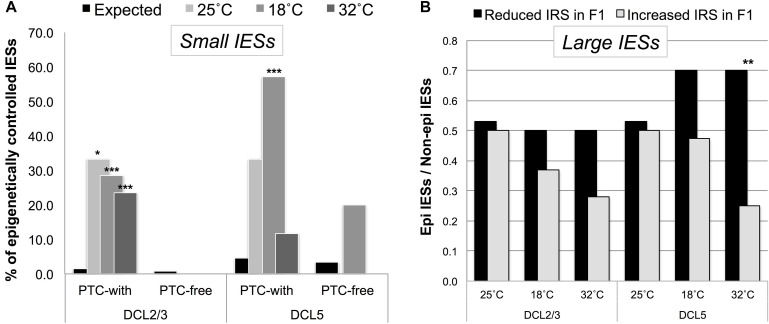
Putative regulated/regulatory IESs fall into a class of IESs that are epigenetically controlled in the *P. tetraurelia* stock 51. **(A)** F1 somatic genomes contain an excess of DCL2/3-sensitive (scnRNA-associated) small exon-mapping and premature-termination codon (PTC)-inducing IESs in genes that are highly expressed in the parental generation. The bars illustrate the fraction of epigenetically regulated IESs in the sets of incompletely excised IESs (IRS > 0.1) detected in F1 cells cultivated at 25, 18, and 32°C (Observed), and in the relevant underlying sub-population of IESs with IRS ≥ 0 (Expected). *P < 0.05; ***P < 0.0001. **(B)** An excess of DCL5-sensitive large exon-mapping IESs (**P < 0.01) undergo reduced levels of incomplete excision [reduced IES Retention Score (IRS)] in F1 cells cultivated at 32°C. The parental F0 cells are shared across all the sets of F1 cells and are cultivated at 25°C.

We found that small PTC-inducing IESs that map to HEG exons preferentially fall into a class of DCL2/3-sensitive IESs (Figure 5A). Instead, large exon-mapping IESs with reduced incomplete excision (i.e., reduced IRS) in F1 cells are enriched with DCL5-sensitive IESs (32°C_*F*1_: Fisher’s Exact Test, *P* = 0.008; Figure 5B). The limited dataset does not allow us to test whether this enrichment is specific to WEG in the parental generation.

We also leveraged the study of epi-IESs (DCL2/3-sensitive IESs, in particular) to rule out an additional interpretation of our observations. Namely, selection may have promoted the gain of PTCs in *Paramecium*’s weakly excised and HEG-mapping IESs to solely deal with the detrimental effects of IES retention, not because IES retention changes the level of gene expression. If selection promoted PTC retention to deal with the detrimental effects of IES incomplete excision, then epi-IESs should be as likely to introduce PTCs as non-epi-IESs are. However, under a model of environmentally induced and IES-mediated modulation of gene expression, epi-IESs should be more likely than non-epi-IESs to contain PTCs. We found that the incomplete excision of non-epi-IESs is 2.4-fold more likely to introduce PTCs in the corresponding transcripts than to leave the ORF intact (PTC-inducing = 348, PTC-free = 147). On the other hand, the incomplete excision of epi-IESs is 14-fold more likely to introduce PTCs (PTC-inducing = 14, PTC-free = 1). This relative enrichment of PTC-inducing epi-IESs holds for 18°C_*F*1_ cells (9 vs. 2.3 fold).

As the bias in favor of PTC-inducing epi-IESs could result from these IESs’ more elevated (and therefore potentially more detrimental) level of retention compared to non-epi-IESs (median IRS: 0.023 vs. 0.013 for epi- and non-epi-IESs, respectively), we reanalyzed our data after accounting for the IRS discrepancy between the two sets of IESs, i.e., upon considering non-epi-IESs with IRS > 0.023. We found that the incomplete excision of non-epi-IESs is still not more than ∼2 fold more likely to introduce PTCs (PTC-inducing = 80, PTC-free = 41). Thus, epi-IESs are substantially more likely than non-epi-IESs to induce PTCs upon their retention.

These findings and the foregoing observations align with a model where PTC induction is environmentally regulated. In this model, (1) the partial retention of scnRNA-associated small IESs may reduce the number of productive gene copies that are highly expressed in the parental generation, whereas (2) the more accurate excision of iesRNA-dependent large IESs may increase the copy number of productive genes that are weakly expressed in the parental generation.

### Developmentally Regulated Biological Functions in *Paramecium*

Next, we asked whether the epigenetically regulated changes in IES recognition/excision efficiency target specific biological functions. We first examined the function of genes that harbor small DCL2/3-sensitive, PTC-inducing, and exon-mapping IESs and are highly expressed at standard conditions (19 genes). Despite this gene set’s very small size, we detected an over-representation of genes involved in aminoacyl-tRNA biosynthesis (Fisher’s exact test, *P* = 0.003). This raises the possibility that PDE may specifically attenuate translation, a classic signature of cellular stress response. We also detected an excess of genes with metalloprotease activity (Fisher’s exact test, *P* < 0.001), which are known to regulate growth factors (Massagué and Pandiella, 1993). If the enhanced retention of small IESs truly reduces the expression of metalloproteases, then one might expect that growth factors’ activation (and thus cell growth) is hindered. It is worth noting that, reduced cell growth in *Paramecium* is coupled with an increased tolerance to stress (Thind et al., 2020).

We then turned to genes that are weakly expressed at standard conditions and that harbor large, DCL5-sensitive, PTC-inducing, and exon-mapping IESs (203 genes). We detected an over-representation of genes encoding proteins that are integral components of membrane (Fisher’s exact test, *P* = 5.3E-10), involved in import, cyclic nucleotide biosynthetic process, and intracellular signal transduction (Fisher’s exact test, *P* < 0.01), metal ion binding and ion channel activity (Fisher’s exact test, *P* < 0.05). Protein domains such as *Insulin-like growth factor binding* and *Epidermal growth factor-like* are amongst the most highly over-represented (Fisher’s exact test, *P* < 8.5E-12).

Taken together, these enrichment analyses indicate that environmentally induced plasticity in *Paramecium* may recalibrate stress sensing and responses to environmental conditions (e.g., nutrients).

### Parental Experience Contributes to Non-random Developmental Plasticity

Finally, we considered whether directional changes in PDE-mediated somatic variation result from conditions that *Paramecium* experiences prior to, rather than during, the phase of somatic development. To gain first insights into this question, we examined the somatic genome of F1 *Paramecium* cells, which were collected in a previous experiment (Vitali et al., 2019) but to date have remained unexplored. Like the F1 lines described above (18°C_*F*1_, 25°C_*F*1_, 32°C_*F*1_), this additional F1 line (henceforth referred to as 25°C^∗^_*F*1_) originates from parental, daily re-isolated cells grown at 25°C. Unlike the other F1 cells, however, the parental line of 25°C^∗^_*F*1_ was additionally subjected to a 40°C heat shock for 30 s daily during vegetative life. Thus, we are in a position to compare the IES retention profile of two sister lines (25°C_*F*1_ and 25°C^∗^_*F*1_) that experienced the same temperature during development (25°C) but originate from isogenic parental cells that experienced different ecological conditions during their vegetative life (constant 25 vs. 25°C with daily 40°C shock for 30 s).

**FIGURE 6:**
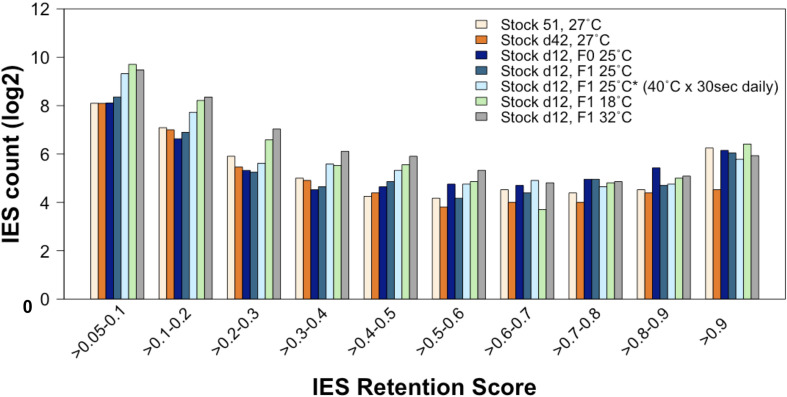
The rate of incomplete IES excision is affected by the ecological conditions to which the parental generation is exposed during vegetative life. Compared to lines that have undergone autogamy at 25–27°C, the count (in log2) of incompletely excised IESs is larger for several IRS classes not only in cells that have undergone autogamy at 18 and 32°C but also in cells that have undergone autogamy at 25°C but have been grown at 25°C and exposed daily to 40°C for 30 s.

First, we compared the incomplete IES excision profiles of the 25°C_*F*0_, 25°C_*F*1_, and 25°C^∗^_*F*1_ mass-cultured cells (Figure 6). Strikingly, this analysis reveals an excess of somatic IESs in the 25°C^∗^_*F*1_ line similar to that reported for the 18°C_*F*1_ and 32°C_*F*1_ lines (Vitali et al., 2019). This observation suggests that the ecological conditions that *Paramecium* cells experience during their vegetative life can impact the phenotype of their sexual offspring.

Further, the count of incompletely excised small and exon-mapping IESs increases for the 25°C^∗^_*F*1_ line as it does for the 18°C_*F*1_ and 32°C_*F*1_ lines, albeit to a lesser extent (Supplementary Figure 1) (F1/F0 ratio: 2.4 and 1.2 for small IESs and large IESs, respectively). 25°C^∗^_*F*1_ cells contain also an excess of incompletely excised small IESs (IRS > 0.1) relative to large IESs in genes that are highly expressed at 25°C (two-proportion Z-test, *P*_*one–tailed*_ = 0.022), with over 80% of these IESs disrupting ORF upon retention. Likewise, the magnitude of incompletely excised large and exon-mapping IESs decreases in the 25°C^∗^_*F*1_ line, as it does for the 18°C_*F*1_ and 32°C_*F*1_ lines (Supplementary Figure 2). The magnitude of the IRS reduction is statistically significant compared to the parental cells cultivated at 25°C (Wilcoxon rank sum test, *P* = 0.0009). There is also a tendency of large exon-mapping IESs with significantly reduced IRS in F1 cells to reside in genes that are weakly expressed in the parental environment (25°C^∗^_*F*1_: 6/6 with reduced IRS vs. 1/2 with increased IRS). These findings suggest that the vegetative life experience of the parental cells contributes to the directionality of the next developmental program.

Finally, we inspected how many IESs with IRS > 0.1 overlap exclusively between the 18°C_*F*1_, 32°C_*F*1_, and 25°C^∗^_*F*1_ lines. We detected 11 somatic IESs, of which 10 small and intragenic (a significant excess compared to large IESs; two-proportion Z-test, *P* = 0.001) (Supplementary Table 1). The 18°C_*F*1_, 32°C_*F*1_, and 25C^∗^_*F*1_ lines share also IESs with significantly reduced IRS (18; 11 intragenic, Supplementary Table 2) and significantly increased IRS (12; 6 intragenic, Supplementary Table 3) relative to the parental line.

These observations suggest that different environmental changes during/prior to somatic development induce non-random molecular dynamics in *Paramecium*. They imply that the newly developing somatic nucleus has access to predictive cues.

## Conclusion

Although adaptive plasticity is currently studied only in multicellular species (Gluckman et al., 2009; Beldade et al., 2011; Bateson et al., 2014; Lea et al., 2017; Lu et al., 2019), plasticity can also unfold in unicellular systems like ciliates, which have a stable differentiation between germ line and soma. Previous studies have shown that the ciliate *Paramecium* experiences plasticity when grown in standard laboratory conditions (Arnaiz et al., 2012; Catania et al., 2013), and more conspicuously upon environmental change (Vitali et al., 2019). Here, we extend our current understanding of the molecular events that unfold during somatic development and the interdependence between these processes and the external environment.

Based on our findings, we propose that alternative DNA-level splicing in *Paramecium* may not only reflect errors or snapshots of a mutual process of germ line/somatic DNA sequence conversion (Arnaiz et al., 2012; Catania et al., 2013, 2020), but also regulatory events that are informed by environmental conditions, in line with previous observations (Nowacki et al., 2010; Cervantes et al., 2013; Singh et al., 2014). In this sense, alternative DNA-level splicing in *Paramecium* is reminiscent of the eukaryotic process of alternative RNA-level splicing (Lewis et al., 2003; Catania and Lynch, 2008; Kelemen et al., 2013; Catania and Schmitz, 2015; Bush et al., 2017; Saudemont et al., 2017).

While waiting for experimental work to determine the hypothesized functional role of IES retention, our observations suggest a cross-generational model for how adaptation to environmental changes may take place in *Paramecium*. Under this model, the exposure of vegetative cells to new environments mediates a reconfiguration of the epigenetic/transcriptional profile, which may improve the fit between these cells’ phenotype and their environment. At the next sexual cycle, this non-genetic reconfiguration recalibrates the trajectory of somatic development, guiding the epigenetic processes that regulate IES excision. This yields a filial somatic macronucleus where non-random developmental plasticity tunes the number of productive somatic gene copies to the new environment, promoting adaptive physiological alterations. Genetic changes that hardwire these epigenetic/physiological changes might follow.

The predictions that this model generates on the relationship between *plasticity* and evolution can be tested in single-celled ciliates as well as in multicellular systems, where germ line sequences that mobilize in response to stress may be viewed as the equivalent of IES retention/excision in *Paramecium*. While the hypothesized adaptive developmental program is conceivably an ancestrally selected feature, there is no reason to expect that it is hindered in genetically homogeneous populations. Another prediction is that the environmental changes that can induce the adaptive developmental program may not have been experienced in the evolutionary past — we deem it unlikely that *Paramecium* experienced daily 30 s shock pulses at 40°C during its evolutionary history. We propose that different changes in the environment are capable of inducing similarly, albeit to different degrees, the adaptive developmental program that evolved to help *Paramecium* cope with changing conditions.

In sum, our findings provide insights into the epigenetic regulation of PDE and *plasticity* in the single-celled *Paramecium*. They suggest that conditions experienced by the previous sexual generation(s) may impact the response to current ecological changes, consistent with previous studies and interpretations in long-lived animals, including humans (Kuzawa, 2005; Zipple et al., 2019). These observations call for future studies to explore models where adaptive phenotypes do not solely result from the interplay between genes and current environment, but also account for the epigenetically controlled transfer of ecological information across generations.

## Data Availability Statement

The datasets presented in this study can be found in online repositories. The names of the repository/repositories and accession number(s) can be found below: https://www.ebi.ac.uk/ena, PRJEB28697, ERR501376, ERR138450, and ERR138952.

## Author Contributions

RH performed the data acquisition, carried out computational analysis, and wrote the manuscript. VV performed the data acquisition and wrote the manuscript. FC conceived the study, carried out computational analysis, supervised, wrote the manuscript, and secured funding. All authors contributed to the article and approved the submitted version.

## Conflict of Interest

The authors declare that the research was conducted in the absence of any commercial or financial relationships that could be construed as a potential conflict of interest.

## Footnotes

1 https://www.ebi.ac.uk/ena
