# Supplementary material for: Cross-Generational Effects and Non-random Developmental Response to Temperature Variation in Paramecium
Source: Front Cell Dev Biol. 2020 Oct 20;8:584219. doi: 10.3389/fcell.2020.584219 (PMC7606892; doi:10.3389/fcell.2020.584219)
Supplement: Supplementary file 1 [file Table_1.DOCX]

Supplementary Material

**
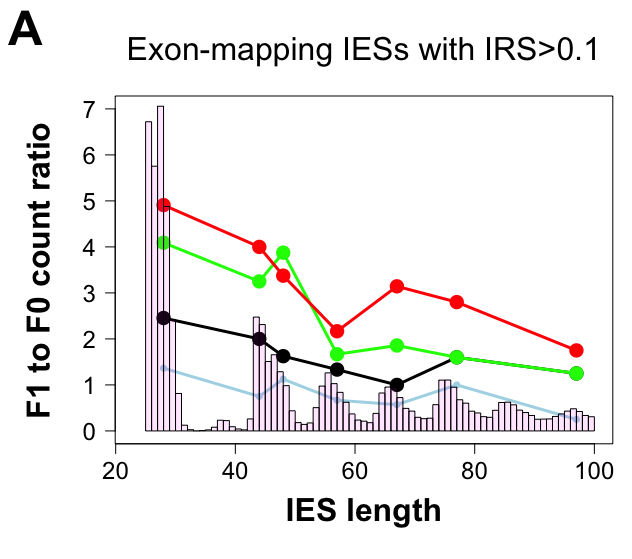

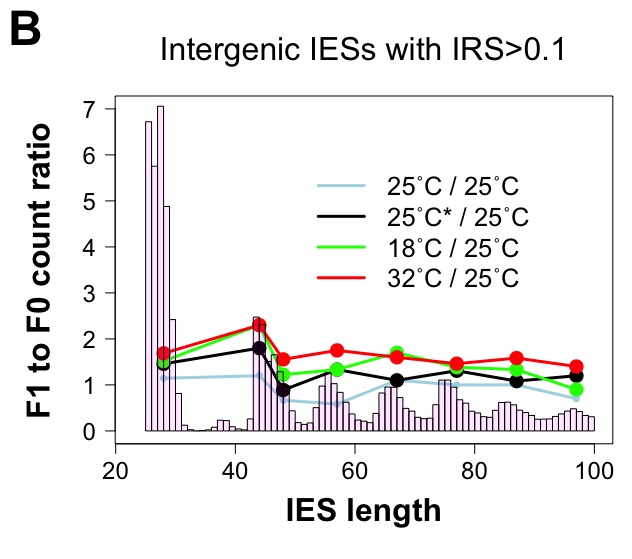
**

**Supplementary Figure 1**. **Ratio of incomplete IES excision (IRS>0.1) counts in parental and filial generations**. The two consecutive generations were exposed to either a stable environment in which both F0 and F1 were cultivated at 25°C (light blue) or a changing environment in which the F0 was cultivated at 25°C, but the F1 was cultivated either at 18°C (green) or 32°C (red), or the F0 was cultivated at 25°C intercalated daily with 30’’ exposure to 40˚C (black line). Grouping IESs according to genomic location (coding exons, intergenic) reveals that small exon-mapping IESs are more often incompletely excised compared to larger exon-mapping IESs **(A)**. No compelling size-dependent pattern is detected for intergenic IESs **(B)**. Ratios were calculated after summing incompletely excised IESs falling in the most frequent size classes (bp): 26-30, 44-45, 46-50, 54-60, 64-70, 74-80, 84-90, and 94-100. Only size classes with ≥3 incompletely excised IESs *per* condition were examined.

**
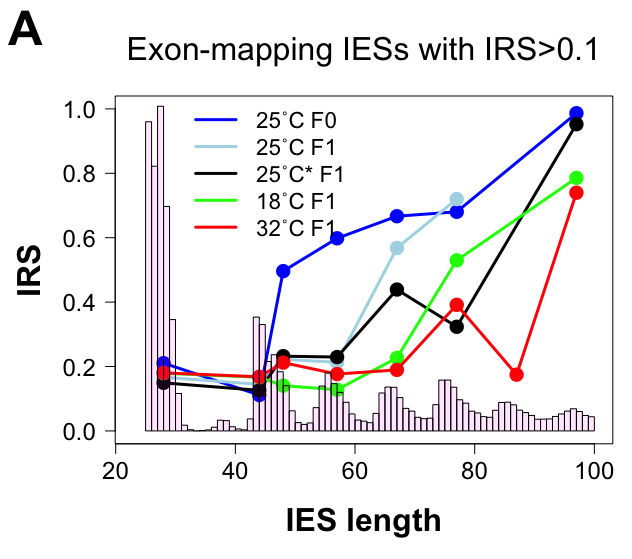

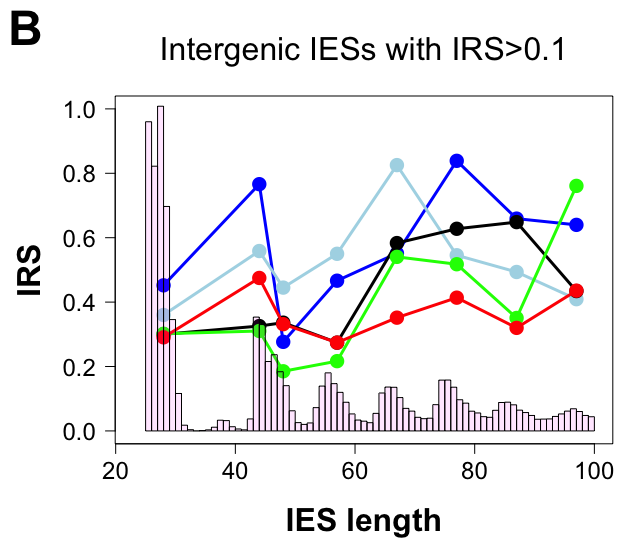
**

**Supplementary Figure 2.** **Relationship between IES size and IES Retention Score (IRS). (A)** The median IES Retention Score (IRS) of small IESs, but not of large IESs, is comparable between F0 and F1 cells cultivated at the standard temperature of 25°C (dark and light blue lines) and at the non-standard culture temperatures of 18°C (green line) and 32°C (red line), and F1 cells cultivated at 25°C and originated from parental cells grown at 25˚C intercalated daily with 30’’ exposure to 40˚C (black line). **(B)** In IESs that map to intergenic regions no clear IES size-related trend is discernible. Median IRS values were calculated using incompletely excised IESs that fall in the most frequent size classes (bp): 26-30, 44-45, 46-50, 54-60, 64-70, 74-80, 84-90, and 94-100. Only size classes with ≥3 incompletely excised IESs *per* condition were examined. Somatic IESs: incompletely excised IESs with IRS>0.1.

**Supplementary Table 1.** Intragenic somatic IESs (IRS>0.1) shared among the 18˚C_F1_, 32˚C_F1_, and 25˚C*_F1_ lines and with IRS≤ 0.1 in the 25˚C_F0_ and 25˚C_F1_ lines. Notes on gene are extracted from ParameciumDB (<https://paramecium.i2bc.paris-saclay.fr/>). *: **Highly expressed genes at 25˚C**.

| **IES ID** | **IES-related gene** | **Notes on gene** |
| --- | --- | --- |
| IESPGM.PTET51.1.102.18202 | PTET.51.1.G1020012 | Cilia- and flagella-associated protein |
| IESPGM.PTET51.1.122.11885 | PTET.51.1.G1220006 | Non-coding gene, almost no expression |
| IESPGM.PTET51.1.142.17798 | PTET.51.1.G1420005 | Stress-inducible protein, putative in *Arabidopsis*; Hsp70/Hsp90 organizing protein (HOP) in *Plasmodium* |
| IESPGM.PTET51.1.155.112078 | **PTET.51.1.G1550065*** | Zinc finger, B-box; highly conserved across *Paramecium* species |
| IESPGM.PTET51.1.160.8661 | **PTET.51.1.G1600009*** | Trans-membrane protein; Variant surface glycoprotein; conserved across *Paramecium* species |
| IESPGM.PTET51.1.2.481827 | PTET.51.1.G0020257 | FCH-domain; conserved across *Paramecium* species |
| IESPGM.PTET51.1.33.200046 | PTET.51.1.G0330134 | C-Jun-amino-terminal kinase-interacting protein; conserved across *Paramecium* species |
| IESPGM.PTET51.1.48.443357 | **PTET.51.1.G0480253*** | Stress-associated endoplasmic reticulum protein 2; conserved across *Paramecium* species |
| IESPGM.PTET51.1.8.9472 | PTET.51.1.G0080008 | Heat shock 70-related protein 5; Exponential increase in expression from veg to dev4 |
| IESPGM.PTET51.1.98.226198 | PTET.51.1.G0980149 | Non-coding gene, almost no expression |
| IESPGM.PTET51.1.193.2264 | PTET.51.1.G1930001 | In *Dictyostelium discoideum* it protects cells against oxidative stress and cell death |

**Supplementary Table 2.** Intragenic somatic IESs shared among the 18˚C_F1_, 32˚C_F1_, and 25˚C*_F1_ lines and with significantly reduced IRS compared to the parental 25˚C_F0_ line. Notes on gene are extracted from ParameciumDB (<https://paramecium.i2bc.paris-saclay.fr/>). *: **Weakly expressed genes at 25˚C**.

| **IES ID** | **IES-related gene** | **Notes on gene** |
| --- | --- | --- |
| IESPGM.PTET51.1.109.145698 | **PTET.51.1.G1090083*** | Up-regulator of cell proliferation |
| IESPGM.PTET51.1.116.113015 | **PTET.51.1.G1160065*** | Aurora kinase; it contributes to the regulation of cell cycle progression |
| IESPGM.PTET51.1.131.255283 | **PTET.51.1.G1310136*** | Non-coding gene |
| IESPGM.PTET51.1.133.30120 | PTET.51.1.G1330020 | Phospholipase D family; Trans-membrane protein; in *Arabidopsis* it regulates vesicle trafficking and auxin responses |
| IESPGM.PTET51.1.134.17823 | **PTET.51.1.G1340015*** | Mitochondrial carrier protein; Trans-membrane protein |
| IESPGM.PTET51.1.140.242472 | **PTET.51.1.G1400158*** | Conserved across *Paramecium* species; Trans-membrane protein; AMP-binding enzyme family protein |
| IESPGM.PTET51.1.161.126708 | **PTET.51.1.G1610085*** | Insulin-like growth factor binding protein, N-terminal; Epidermal growth factor-like domain; Trans-membrane protein; Conserved across *Paramecium* species; |
| IESPGM.PTET51.1.33.125737 | **PTET.51.1.G0330080*** | Insulin-like growth factor binding protein, N-terminal; Epidermal growth factor-like domain; Trans-membrane protein; Proprotein convertase subtilisin/kexin type 5 |
| IESPGM.PTET51.1.50.285016 | PTET.51.1.G0500179 | Pectinacetylesterase |
| IESPGM.PTET51.1.69.379537 | **PTET.51.1.G0690203*** | Non-coding gene |
| IESPGM.PTET51.1.92.7200 | **PTET.51.1.G0920001*** | In *Prochlorococcus marinus* has a central role in coupling the hydrolysis of ATP to the transfer of proteins into and across the cell membrane, serving as an ATP-driven molecular motor driving the stepwise translocation of polypeptide chains across the membrane |

**Supplementary Table 3.** Intragenic somatic IESs shared among the 18˚C_F1_, 32˚C_F1_, and 25˚C*_F1_ lines and with significantly increased IRS compared to the parental 25˚C_F0_ line. Notes on gene are extracted from ParameciumDB (<https://paramecium.i2bc.paris-saclay.fr/>). *: **Highly expressed genes at 25˚C**.

| **IES ID** | **IES-related gene** | **Notes on gene** |
| --- | --- | --- |
| IESPGM.PTET51.1.131.10969 | PTET.51.1.G1310006 | It may be involved in the pathway protein glycosylation, which is part of protein modification. |
| IESPGM.PTET51.1.1.358366 | **PTET.51.1.G0010219*** | In plants it is involved in the responding to wounding or pathogen challenge by the increased formation of cell wall-bound ferulic acid polymers |
| IESPGM.PTET51.1.142.19468 | PTET.51.1.G1420005 | Stress-inducible protein, putative |
| IESPGM.PTET51.1.155.112078 | **PTET.51.1.G1550065*** | It can regulate their cellular processes via cross-talk between glycosylation and phosphorylation or by affecting proteolytic processing |
| IESPGM.PTET51.1.312.4680 | PTET.51.1.G3120004 | It is involved in the pathway protein glycosylation |
| IESPGM.PTET51.1.76.99536 | PTET.51.1.G0760058 | Trans-membrane helix prediction; Required for cellular homeostasis and for survival from DNA damage and developmental changes induced by stress |
